# Functional MRI-Specific Alterations in Salience Network in Mild Cognitive Impairment: An ALE Meta-Analysis

**DOI:** 10.3389/fnagi.2021.695210

**Published:** 2021-07-26

**Authors:** Yu Song, Wenwen Xu, Shanshan Chen, Guanjie Hu, Honglin Ge, Chen Xue, Wenzhang Qi, Xingjian Lin, Jiu Chen

**Affiliations:** ^1^Department of Neurology, The Affiliated Brain Hospital of Nanjing Medical University, Nanjing, China; ^2^Institute of Neuropsychiatry, The Affiliated Brain Hospital of Nanjing Medical University, Nanjing, China; ^3^Institute of Brain Functional Imaging, Nanjing Medical University, Nanjing, China; ^4^Department of Radiology, The Affiliated Brain Hospital of Nanjing Medical University, Nanjing, China

**Keywords:** regional homogeneity, mild cognitive impairment, salience network, amnestic mild cognitive impairment, amplitude of low-frequency fluctuation, functional connectivity, activation likelihood estimation

## Abstract

**Background** Mild cognitive impairment (MCI) is an intermediate stage between normal aging and dementia. Amnestic MCI (aMCI) and non-amnestic MCI are the two subtypes of MCI with the former having a higher risk for progressing to Alzheimer's disease (AD). Compared with healthy elderly adults, individuals with MCI have specific functional alterations in the salience network (SN). However, no consistent results are documenting these changes. This meta-analysis aimed to investigate the specific functional alterations in the SN in MCI and aMCI.

**Methods:** We systematically searched PubMed, Embase, and Web of Science for scientific neuroimaging literature based on three research methods, namely, functional connectivity (FC), regional homogeneity (ReHo), and the amplitude of low-frequency fluctuation or fractional amplitude of low-frequency fluctuation (ALFF/fALFF). Then, we conducted the coordinate-based meta-analysis by using the activation likelihood estimation algorithm.

**Results:** In total, 30 functional neuroimaging studies were included. After extracting the data and analyzing it, we obtained specific changes in some brain regions in the SN including decreased ALFF/fALFF in the left superior temporal gyrus, the insula, the precentral gyrus, and the precuneus in MCI and aMCI; increased FC in the thalamus, the caudate, the superior temporal gyrus, the insula, and the cingulate gyrus in MCI; and decreased ReHo in the anterior cingulate gyrus in aMCI. In addition, as to FC, interactions of the SN with other networks including the default mode network and the executive control network were also observed mainly in the middle frontal gyrus and superior frontal gyrus in MCI and inferior frontal gyrus in aMCI.

**Conclusions:** Specific functional alternations in the SN and interactions of the SN with other networks in MCI could be useful as potential imaging biomarkers for MCI or aMCI. Meanwhile, it provided a new insight in predicting the progression of health to MCI or aMCI and novel targets for proper intervention to delay the progression.

**Systematic Review Registration:** [PROSPERO], identifier [No. CRD42020216259].

## Introduction

Mild cognitive impairment (MCI) is universally considered as the predementia phase of Alzheimer's disease (AD) (Gao et al., 2020). About one in five community-dwelling elders aged 65 years and above suffer from MCI (Livingston et al., 2017). MCI has been divided into two subtypes, amnestic MCI (aMCI) and non-amnestic MCI (naMCI), according to the difference of impaired cognitive domain. The former manifests as memory deficit combined with or without slight impairment of other cognitive domains and converts to AD at a high rate of 10–15% per annum (Cai et al., 2020a). Some researchers have estimated that the number of patients with AD would be reduced by half if the onset of AD is delayed by five years (Butterfield and Boyd-Kimball, 2018). Furthermore, accurate diagnosis of the early or preclinical stage of AD can provide a basis for early intervention. Therefore, determining the specific change of MCI or aMCI is important. Studies have used the resting-state functional magnetic resonance imaging (rs-fMRI) for investigating neuronal integrity and functional alteration in the brain of patients with MCI (Khazaee et al., 2016; Zhu et al., 2016). Salience network (SN), which is involved in affections, attention, emotions, and switching between cognitive resources, is significantly affected in patients with MCI (Chen H. et al., 2019). Many studies have reported functional alterations in the SN in MCI when compared with healthy controls (HCs). However, there were no consistent results (Chen J. et al., 2019). Thus, our study aimed to conclude the characteristic abnormalities of the SN in MCI by analyzing previous investigations.

The rs-fMRI has been widely used to detect functional changes, such as disruptions in network integrity in the brain, and to predict future cognitive decline in normal elderly participants or MCI (Kaiser et al., 2015; Buckley et al., 2017). Functional connectivity (FC), the amplitude of low-frequency fluctuation (ALFF) or fractional amplitude of low-frequency fluctuation (fALFF), and regional homogeneity (ReHo) are the three main methods used for measuring functional changes (Harrington et al., 2017; Cheng et al., 2019). FC, based on independent component analysis (ICA) or seed regions of interest (seed-ROI) analysis, reflects the functional relationship between two or more regions of the brain (Mamah et al., 2013; Chand et al., 2017). ALFF and fALFF are used for quantitatively assessing the total voxel-wise amplitude of the local features of the brain oscillatory activities. The latter is more specific because its measure includes the entire frequency range, and it attenuates the influence of physiological noise (Han et al., 2011; Qing et al., 2017). ReHo, which is based on the similarity of neighboring voxels over a time series, can be used to determine the functional change of the local brain area. It is highly reliable for studying the consistency of local brain activity (Zang et al., 2004; Li M. G. et al., 2020). Thus, the three methods mentioned above have their virtues in investigating brain networks to understand the functional changes in MCI.

The SN, as one of the three core neurocognitive networks [the default mode network (DMN), SN, and the executive control network (ECN)], plays a vital role in cognitive function. It is a task-positive network, serving to identify relevant stimuli for guiding behavior in response to several internal and external stimuli, and is relevant for attention and the interoceptive process (Menon, 2011; Supekar et al., 2019). Many studies have reported functional changes of the SN in patients with cognitive impairment (Agosta et al., 2012; Onoda et al., 2012). In addition, multiple studies have examined the relationships between SN and other networks. Li et al. found that the SN-centered “triple-network model” was impaired in patients with cognitive decline by comparing 33 AD, 24 late MCI, and 25 control subjects (Li et al., 2019). However, the lack of data makes it difficult to find reliable specific imaging markers in the SN to predict the transformation from healthy elders to MCI or MCI to AD. Thus, concluding consistent results of alterations in the SN is essential.

Activation likelihood estimation (ALE) technique is a powerful meta-analysis method based on random-effects interference and controlling for sample size, which uses peak coordinates reported in selected studies as Gaussian probability distributions (Kollndorfer et al., 2013). It avoids the shortcoming of uncertainty of the actual abnormal position by using the coordinates of abnormal sites instead of area label (Hétu et al., 2013). A study by Chen et al. analyzed the structural brain changes in MCI using ALE and suggested that regional alternations might serve as early predictive or diagnostic biomarkers for MCI (Chen et al., 2020). Nevertheless, this study was the first one to assess functional specific changes in the SN in patients with MCI and aMCI.

Our study aimed to comprehensively analyze and conclude the specific functional alterations of brain areas in the SN and discuss the specific abnormal markers within the SN as well as its interaction with other networks in MCI. We proposed the hypothesis that (1) the three indexes (i.e., ALFF/fALFF, ReHo, and FC) of the SN would show specific neuroimaging changes in some brain regions, (2) specific changes would be different in MCI and aMCI, and (3) interactions between SN and other networks could be observed in MCI and aMCI. A detailed understanding of the specific functional alterations of the SN in MCI will provide a more profound understanding of the pathological mechanism of such patients and may help better establish rehabilitation procedures along those lines.

## Materials and Methods

### Search Strategy

A systematic research of studies was conducted according to the Preferred Reporting Items for Systematic Reviews and Meta-Analysis (PRISMA) statement and then recorded using the suggested checklist. We systematically searched PubMed, Web of Science, and Embase for rs-fMRI scientific literature published in English between January 1, 2000 and April 1, 2021. Search keywords were as follows: (1) (salience network) AND (“mild cognitive impairment” [MeSH]) AND (“functional magnetic resonance imaging” [MeSH]) AND (Resting state) AND [(functional connectivity) OR (fc)], (2) (“mild cognitive impairment” [MeSH]) AND (“functional magnetic resonance imaging” [MeSH]) AND (Resting state) AND [(regional homogeneity) OR (ReHo)] OR (local consistency), (3) (“mild cognitive impairment” [MeSH]) AND (“functional magnetic resonance imaging” [MeSH]) AND (Resting state) AND [(amplitude of low frequency fluctuations) OR (ALFF)], and (4) (“mild cognitive impairment” [MeSH]) AND (“functional magnetic resonance imaging” [MeSH]) AND (Resting state) AND [(fractional amplitude of low frequency fluctuations) OR (fALFF)].

**Figure 1:**
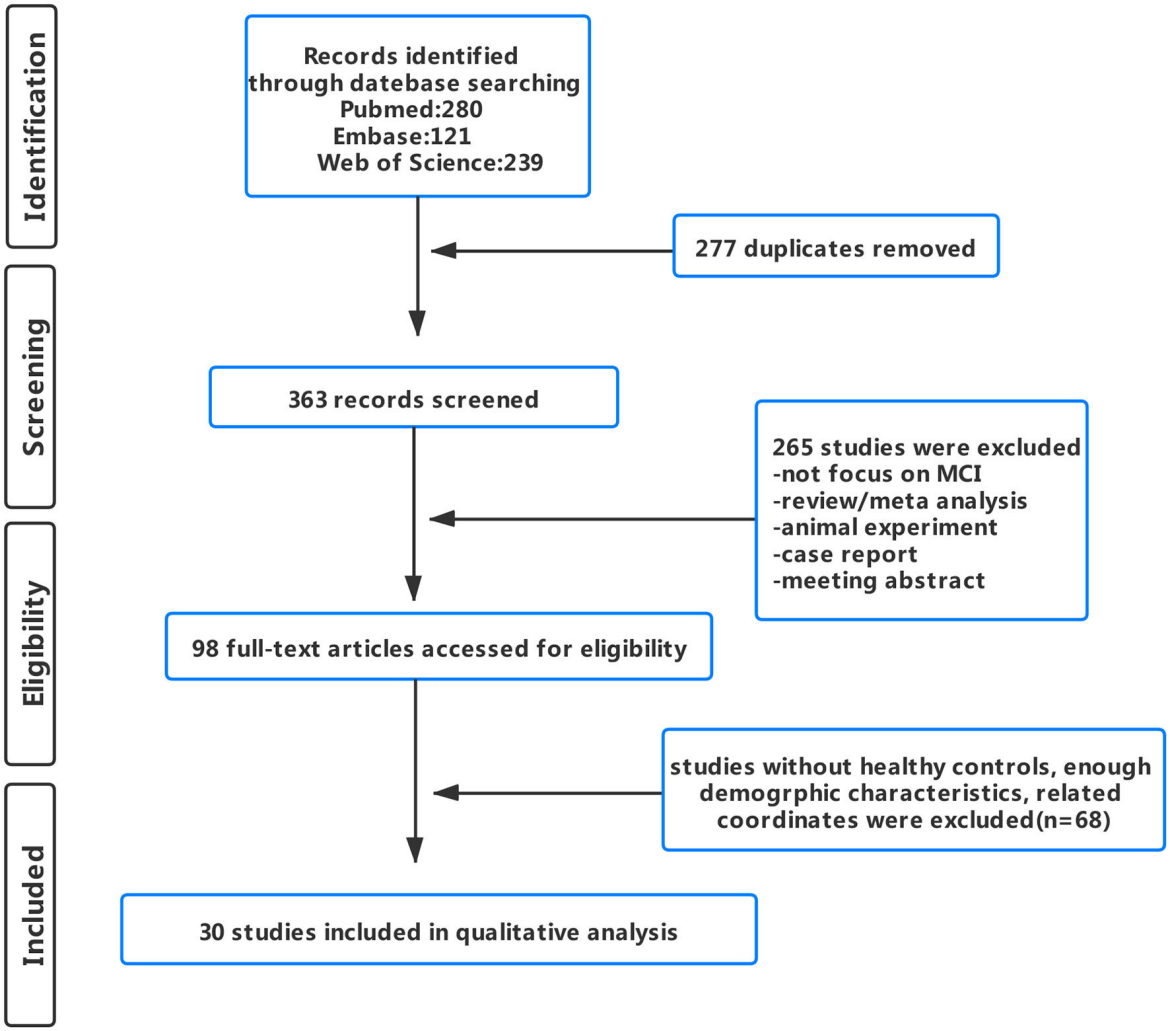
Flowchart shows study selection process.

A total of 353 publications were initially retrieved. After careful screening, a total of 30 publications (7 FC, 13 ALFF/fALFF, 8 ReHo, and 2 ALFF/fALFF & ReHo) were included in the final analysis (Figure 1). Among the included studies, a total of 18 studies met the criteria of aMCI (4 FC, 8 ALFF/fALFF, 5 ReHo, and 1 ALFF/fALFF & ReHo). However, mainly due to the absence of detailed information on neuropsychological assessment, no study met the criteria of naMCI.

### Inclusion and Exclusion Criteria

Studies related to rs-fMRI investigations on MCI were included if they (1) reported significant alterations of FC, ALFF/fALFF, or ReHo in the SN using thresholds uncorrected with spatial extent thresholds or corrected for multiple comparisons; (2) made comparisons between MCI and HCs; (3) reported information about the stereotactic space in Talairach or Montreal Neurologic Institute (MNI) coordinates; and (4) were published in English in peer-reviewed journals. The patients with MCI met the following criteria: (a) evidence of the complaint of cognitive change, (b) impairment in one or more cognitive domains, (c) preservation of the independence of function in daily life, and (d) not demented (Albert et al., 2011). The MCI group was further divided into two types, namely, the aMCI group and the naMCI group, based on whether the cognitive impairment was dominated by memory (Cooper et al., 2015). Only the study with the largest sample size, to avoid repeat count, was included.

Studies investigating patients with MCI with a history of other diseases, such as Parkinson's disease, depression, focal epilepsy, and stroke, were excluded unless they included the group of MCIs with no other concurrent diseases. Meta-analysis, reviews, animal experiments, conference meeting abstracts, case reports, studies without group-level statistics, and articles lacking necessary baseline information were not included in the analysis.

Moreover, the quality of the individual studies was evaluated using the Newcastle–Ottawa Scale (NOS) by two reviewers. Three major domains (i.e., selection, comparability, and the ascertainment of outcome) were included in this scale rates (Lo et al., 2014). At any point, any disagreement between reviewers was resolved by means of meeting and discussion among all authors to establish a consensus.

### Data Extraction

Two researchers independently conducted the data extraction and then checked the accuracy of extracted data. When disagreements appeared, a third reviewer participated in the discussion until a consensus was reached.

In terms of studies focused on FC, studies investigating the FC within the SN or intra-FC between SN and other networks such as the DMN and ECN were all included, and the coordinate of brain regions that revealed significant changes was extracted. Due to the limited number of included studies, we did not further group according to the difference of methods.

In terms of studies focused on ALFF/fALFF and ReHo, which were voxel-wise whole-brain indexes, first, we reviewed all the studies investigating functional alterations in MCI based on ICA or seed ROI to summarize brain areas belong to the SN. According to the results, we extracted the coordinates of brain regions in the SN which revealed significant changes.

### Data Analysis Procedure

The results of the three different methods were divided into increased group and decreased group: (a) MCI: increased ALFF/ fALFF (168 subjects, seven foci, six experiments); decreased ALFF/fALFF (321 subjects, 21 foci, 12 experiments); increased ReHo (166 subjects, eight foci, seven experiments); decreased ReHo (138 subjects, six foci, five experiments); increased FC (111 subjects, 15 foci, four experiments); decreased FC (63 subjects, 26 foci, four experiments). (b) aMCI: increased ALFF/fALFF (126 subjects, four foci, four experiments); decreased ALFF/fALFF (236 subjects, 16 foci, eight experiments); increased ReHo (78 subjects, four foci, three experiments); decreased ReHo (126 subjects, five foci, four experiments); increased FC (30 subjects, one foci, one experiments); decreased FC (47 subjects, five foci, three experiments).

Data were calculated using a Java-based version of Ginger ALE 2.3.6 (http://www.brainmap.org/ale) (Browndyke et al., 2013). Ginger ALE determined whether there was anatomical or functional convergence of differences among multiple coordinate-based human brain imaging studies (Mar, 2011). After transforming Talairach coordinates into MNI space, we sorted all the foci and basic information into six text files. These files were subsequently imported into the software to read the foci. The cluster-forming threshold at *p* < 0.01 was set to obtain the ALE map with a cluster-level family-wise error (FWE) correction at *p* < 0.05 and 1,000 permutations (Eickhoff et al., 2012).

However, among the studies included in the analysis, there was one study that reported increased FC of the SN of the left anterior insula in aMCI, compared with HCs. Only one coordinate was available, and thus, the ALE analysis was not conducted in this group (Li X. et al., 2020).

Following the ALE analysis, a jackknife sensitivity analysis was performed by iteratively repeating the same analysis, excluding one dataset each time to test the replicability of results across studies (Lyles and Lin, 2010). However, due to the limitation of the number of included studies involving different indicators, the sensitivity analysis was performed only on the group of decreased ALFF/fALFF in MCI (a total of 12 included studies).

## Results

### Search Results

Details about the demographics and characteristics of the included studies are summarized in Table 1. The quality assessment of the included studies ranged from low scores of 5 and moderate scores of 6–7 to high scores of 8. The result of the quality assessment is available in the Supplementary Material.

**Table 1:** Demographic characteristics of the included rs-fMRI studies.

**Number**	**Study**	**Sample size(n)**	**Gender (M/F)**	**Age (years ± SD)**	**MMSE (SD)**	**Reference space**	**Group contrasts**	**Foci (n)**	**Threshold**	**Method of analysis**
**ALFF/fALFF**										
1	Cai et al. (2017)	MCI 39	19/20	72.4 ± 5.01	25.51 ± 2.88	MNI	MCI>HC	0	*p* < 0.05 cor	
		HC 38	19/19	73.92 ± 3.90	29.28 ± 0.88		MCI < HC	3		
2	Cha et al. (2015)	MCI 34	18/16	68.4 ± 7.9	27.1 ± 2.	1 MNI	MCI>HC	0	*p* < 0.05 cor	
		HC 62	17/45	68.5 ± 8.0	28.6 ± 1.9		MCI < HC	4		
3	Jia et al. (2015)	MCI 7	2/5	74.1 ± 7.8	27.0 ± 2.3	MNI	MCI>HC	0	*p* < 0.05 cor	
		HC 15	8/7	70.2 ± 7.1	29.2 ± 1.3		MCI < HC	1		
4	Liang et al. (2014)	MCI 53	31/22	73.2 ± 7.3	27.1 ± 2.3	MNI	MCI>HC	0	*p* < 0.05 cor	
		HC 35	18/17	74.3 ± 5.9	28.9 ± 1.6		MCI < HC	1		
5	Liu X. et al. (2014)	MCI 25	16/9	71.88 ± 4.09	27	MNI	MCI>HC	0	*p* < 0.05 cor	
		HC 21	14/7	72.95 ± 3.53	29		MCI < HC	1		
6	Li et al. (2017a)	MCI 27	13/14	67.44 ± 8.49	23.52 ± 3.31	MNI	MCI < HC	0	*p* < 0.05 cor	
		HC 32	16/16	64.88 ± 7.54	27.67 ± 1.67		MCI < HC	2		
7	Ni et al. (2016)	MCI 26	12/14	71 ± 9	25 ± 1.48	MNI	MCI>HC	2	*p* < 0.01 cor	
		HC 28	17/11	70 ± 9	29 ± 1.09		MCI < HC	0		
8	Wang et al. (2011)	MCI 16	7/9	69.38 ± 7.00	26.50 ± 1.03	MNI	MCI>HC	1	*p* < 0.05 cor	
		HC 22	7/15	66.55 ± 7.67	28.59 ± 0.59		MCI < HC	1		
9	Yang et al. (2018)	MCI 55	27/28	67.51 ± 9.62	24.66 ± 4.20	MNI	MCI>HC	1	*p* < 0.001 uncor	
		HC 57	22/35	63.77 ± 8.09	28.14 ± 2.13		MCI < HC	2		
10	Yin et al. (2014)	MCI 11	2/9	66.6 ± 8.7	24.6 ± 3.2	MNI	MCI>HC	0	*p* < 0.05 cor	
		HC 22	12/10	62.1 ± 8.1	29.2 ± 1.1		MCI < HC	2		
11	Zhao et al. (2014)	MCI 20	12/8	65.11 ± 9.92	25.21 ± 2.24	MNI	MCI>HC	1	*p* < 0.01 cor	
		HC 18	10/8	66.81 ± 7.43	29.31 ± 1.22		MCI < HC	2		
12	Zhuang et al. (2019)	MCI 35	23/12	71.42 ± 4.39	27.61 ± 2.14	MNI	MCI>HC	0	*p* < 0.05 cor	
		HC 26	16/10	70.01 ± 5.58	28.51 ± 1.32		MCI < HC	1		
13	Zhuang et al. (2012)	MCI 47	28/19	71.96 ± 4.78	26.98 ± 1.53	MNI	MCI>HC	0	*p* < 0.05 cor	
		HC 33	18/15	72.85 ± 3.39	28.18 ± 1.33		MCI < HC	1		
14	Zhou et al. (2020)	MCI 24	10/14	69.8 ± 6.2	23.9 ± 3.6	MNI	MCI>HC	1	*p* < 0.001 uncor	
		HC 32	14/18	67.9 ± 6.4	28.0 ± 1.9		MCI < HC	0		
15	Zhuang et al. (2020)	MCI 43	18/25	64.50 ± 5.64	–	MNI	MCI>HC	1	*p* < 0.05 cor	
		HC 29	7/22	66.79 ± 3.68	–		MCI < HC	0		
**ReHo**										
1	Bai et al. (2008)	MCI 20	10/10	71.3 ± 3.8	27.2 ± 1.6	Talairac	MCI>HC	0	*p* < 0.005 cor	
		HC 20	9/11	69.4 ± 3.8	28.3 ± 1.4		MCI < HC	1		
2	Cai et al. (2018)	MCI 20	11/9	69.76 ± 6.48	25.61 ± 2.67	MNI	MCI>HC	1	*p* < 0.01 cor	
		HC 53	29/24	76.08 ± 6.45	28.2 ± 2.13		MCI < HC	0		
3	Cha et al. (2015)	MCI 34	18/16	68.4 ± 7.9	27.1 ± 2.1	MNI	MCI>HC	0	*p* < 0.05 cor	
		HC 62	17/45	68.5 ± 8.0	28.6 ± 1.9		MCI < HC	1		
4	Liu Z. et al. (2014)	MCI 12	1/11	59.3 ± 3.3	26.4 ± 0.9	MNI	MCI>HC	1	*p* < 0.01 cor	
		HC 12	4/8	60.6 ± 5.8	29.8 ± 0.4		MCI < HC	0		
5	Luo et al. (2018)	MCI 32	17/15	72.43 ± 4.25	28.34 ± 1.68	MNI	MCI>HC	1	*p* < 0.01 cor	
		HC 49	18/31	73.33 ± 4.60	29.02 ± 1.20		MCI < HC	0		
6	Min et al. (2019)	MCI 10	5/5	69.80 ± 2.66	25.90 ± 0.74	MNI	MCI>HC	2	*p* < 0.05 cor	
		HC 10	5/5	69.90 ± 2.60	29.30 ± 0.83		MCI < HC	0		
7	Ni et al. (2016)	MCI 26	12/14	71 ± 9	25 ± 1.48	MNI	MCI>HC	1	*p* < 0.05 cor	
		HC 28	17/11	70 ± 9	29 ± 1.09		MCI < HC	0		
8	Wang et al. (2015)	MCI 30	18/12	69.1 ± 5.8	26.2 ± 2.2	MNI	MCI>HC	1	*p* < 0.01 cor	
		HC 32	15/17	70.1 ± 5.5	28.1 ± 1.5		MCI < HC	0		
9	Yuan X. et al. (2016)	MCI 36	17/19	66.8 ± 9.5	24.9 ± 3.4	MNI	MCI>HC	1	*p* < 0.01 cor	
		HC 46	19/2	7 64.3 ± 7.8	28.5 ± 2.0		MCI < HC	1		
10	Zhang et al. (2010)	MCI 48	–	72.04 ± 4.42	27	MNI	MCI>HC	0	*p* < 0.05 cor	
		HC 36		71.64 ± 3.72	29		MCI < HC	2		
**FC**										
1	Conwell et al. (2018)	MCI 15	6/9	71.1 ± 6.0	25.0 ± 3.4	MNI	MCI>HC	1	*p* < 0.001 uncor	ICA
		HC 15	6/9	67.3 ± 8.4	29.5 ± 0.6		MCI < HC	0		
2	He et al. (2014)	MCI 18	10/8	70.2 ± 7.9	21.9 ± 5.0	MNI	MCI>HC	0	*p* < 0.05 cor	ICA, Seed ROI
		HC 21	1/14	65.0 ± 8.2	28.5 ± 1.4		MCI < HC	5		
3	Liang et al. (2012)	MCI 16	6/10	68.50 ± 7.77	25.94 ± 1.65	MNI	MCI>HC	10	*p* < 0.05 cor	Seed ROI
		HC 16	6/10	67.19 ± 8.38	28.56 ± 0.63		MCI < HC	15		
4	Li X. et al. (2020)	MCI 30	13/17	68.53 ± 2.97	25.10 ± 0.66	MNI	MCI>HC	1	*p* < 0.001 uncor	ICA
		HC 30	14/16	68.67 ± 3.19	28.20 ± 0.92	MCI < HC	0			
5	Sarli et al. (2021)	MCI 50	25/25	67.13 ± 9.74	21.1 ± 4.89	MNI	MCI>HC MCI < HC	3	*p* < 0.001 uncor	ICA, Seed ROI
		HC 50	30.20	67.14 ± 9.71	28.12 ± 1.57		0			
6	Yi et al. (2015)	MCI 10	2/8	70.70 ± 1.71	23.70 ± 0.87	MNI152	MCI>HC	0	*p* < 0.005 uncor	ICA
		HC 12	3/9	71.75 ± 1.21	27.40 ± 0.45		MCI < HC	1		
7	Zhu et al. (2016)	MCI 19	7/12	65.7 ± 10.7	26.7 ± 1.6	MNI	MCI>HC	0	*p* < 0.05 cor	Seed ROI
		HC 28	11/17	63.8 ± 6.7	29.0 ± 0.8		MCI < HC	3		

*rs-fMRI, resting-state functional magnetic resonance imaging; MNI, Montreal Neurologic Institute; MCI, mild cognitive impairment; HCs, healthy controls; SD, standard deviation; ALFF/fALFF, the amplitude of low frequency fluctuation/fractional amplitude of low-frequency fluctuation; ReHo, regional homogeneity; FC, functional connectivity; MMSE. Mini-mental state examination; ICA, independent component analysis; Seed ROI, seed regions of interest*.

The brain areas of the SN were summarized as follows: (1) Use ICA: bilateral anterior insula (Yi et al., 2015), bilateral frontoinsular cortex, bilateral anterior cingulate cortex (ACC) (Han et al., 2011), bilateral dorsal ACC (dACC), bilateral insula lobule (Chand et al., 2017), right supplementary motor area (SMA), left superior temporal gyrus (STG), bilateral supramarginal gyrus (SG), left precuneus (Conwell et al., 2018; Cai et al., 2020b), preSMA (Yi et al., 2015), dorsal anterior cingulate cortex/medial prefrontal cortex (dACC/MPFC), dorsolateral prefrontal cortex (DLPFC) (He et al., 2014), bilateral caudate (Agosta et al., 2012), the thalamus (Wang et al., 2020), bilateral ventrolateral prefrontal cortex, bilateral STG/insula, midcingulate cortex/preSMA, medial orbitofrontal cortex, and bilateral orbital/frontoinsular (Agosta et al., 2012) and (2) Use seed ROI: bilateral frontoinsular cortex (Joo et al., 2017), bilateral anterior insula, bilateral dACC, dACC/MPFC, bilateral anterior insula (Zhu et al., 2016), bilateral insula (Sarli et al., 2021), and bilateral SG (Liang et al., 2012).

### Meta-analysis Results

#### Regions With Significant Changes Between MCI and HCs

**Figure 2:**
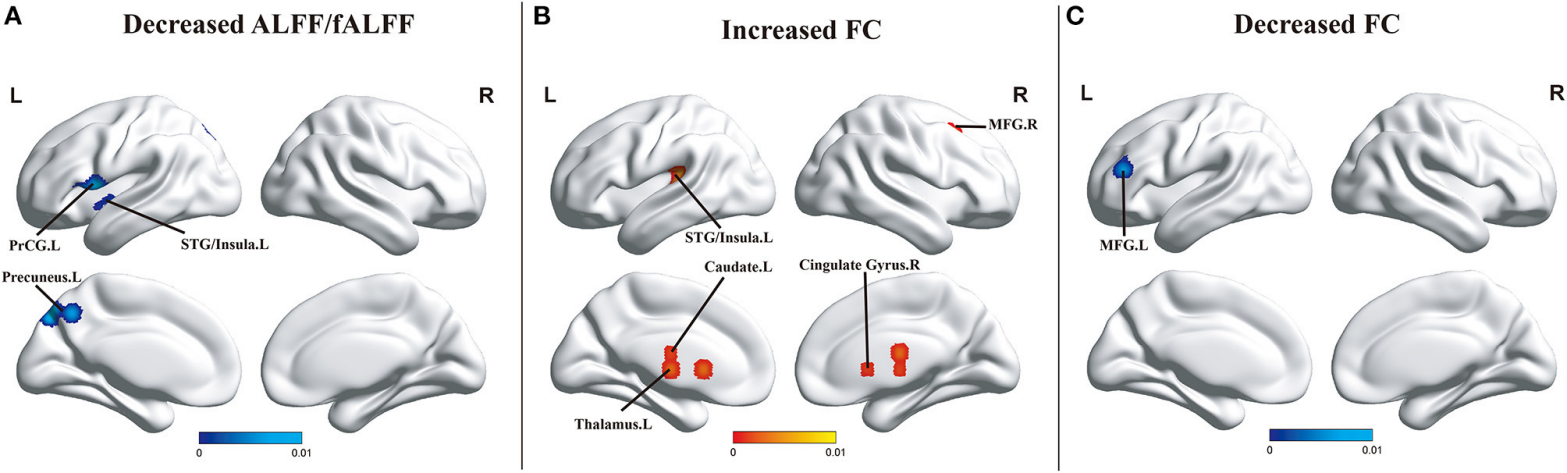
**(A)** Brain regions showing decreased ALFF/fALFF in patients with MCI compared with HCs. **(B)** Brain regions showing increased FC in patients with MCI when compared with HCs. **(C)** Brain regions showing decreased FC in patients with MCI compared with HCs. The color bar represents the *p*-value. Areas with decreased change relative to controls are displayed in blue, and areas with increased change are displayed in red. Results are thresholded at *p* < 0.01 cluster-corrected and *p* < 0.05 family-wise error corrected. Abbreviations: MCI, mild cognitive impairment; HCs, healthy controls; ALFF/fALFF, the amplitude of low-frequency fluctuation/fractional amplitude of low-frequency fluctuation; FC, functional connectivity; MFG, middle frontal gyrus; PreCG, precentral gyrus; STG, superior temporal gyrus; R, right; L, left.

Compared with HCs, patients with MCI showed decreased ALFF/fALFF in the left STG (BA 22), left insula (BA 13), left precentral gyrus (PreCG/BA 44), and the precuneus (BA 7). No brain region presenting increased ALFF/fALFF was found. In addition, patients with MCI showed increased FC of the SN in the thalamus, the caudate, the cingulate gyrus (BA 32), the right middle frontal gyrus (MFG/BA 41), and the left STG/insula (BA 41) and decreased FC in the left MFG (BA 46) and superior frontal gyrus (SFG/BA 9). However, no brain area presenting increased ReHo or decreased ReHo was observed (Figure 2).

#### Regions With Significant Changes Between aMCI and HCs

**Figure 3:**
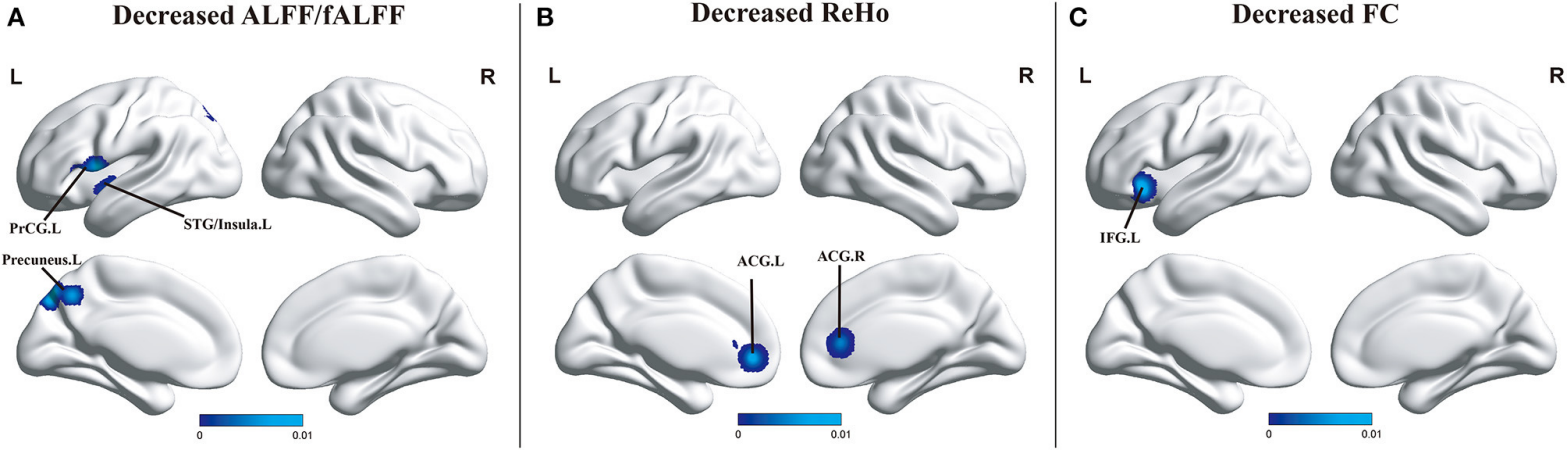
**(A)** Brain regions showing decreased ALFF/fALFF in patients with aMCI compared with HCs. **(B)** Brain regions showing decreased ReHo in patients with aMCI compared with HCs. **(C)** Brain regions showing decreased FC in patients with aMCI when compared with HCs. The color bar represents the *p*-value. Areas with decreased change relative to controls are displayed in blue, and areas with increased change are displayed in red. Results are thresholded at *p* < 0.01 cluster-corrected and *p* < 0.05 family-wise error corrected. Abbreviations: aMCI, amnestic mild cognitive impairment; HCs, healthy controls; ALFF/fALFF, the amplitude of low-frequency fluctuation/fractional amplitude of low-frequency fluctuation; ReHo, regional homogeneity; FC, functional connectivity; ACG, anterior cingulate gyrus; PreCG, precentral gyrus; STG, superior temporal gyrus; IFG, inferior frontal gyrus; R, right; L, left.

Compared with HCs, patients with aMCI revealed decreased ALFF/fALFF in the left STG (BA 22), left insula (BA 13), left PreCG (BA 44), and the precuneus (BA 7), and no brain region presenting increased ALFF/fALFF was observed, which was in line with what is seen in MCI. Differently, patients with aMCI showed decreased FC in the left inferior frontal gyrus (IFG) and decreased ReHo in the anterior cingulate gyrus (ACG). However, no brain area presenting increased FC or increased ReHo was observed (Figure 3).

More details about clusters from the ALE analysis are summarized in Table 2.

**Table 2:** All clusters from the ALE analysis.

**Cluster**	**Volume (mm^**3**^)**	**MNI**			**Maximum ALE value**	***Z* value**	**Side**	**Anatomical regions**	**BA**
		**X**	**Y**	**Z**					
ALFF/fALFF									
MCI>HC									
NA									
MCI < HC									
1	5,720	−56	8	0	0.009969907	4.2067027	Left	Superior temporal gyrus	22
1	5,720	−54	6	8	0.009410367	4.0713005	Left	Precentral gyrus	44
1	5,720	−52	18	2	0.008123473	3.4053626	Left	Precentral gyrus	44
1	5,720	−46	6	−6	0.00798626	3.3795779	Left	Insula	13
1	5,720	−46	−2	−2	0.007434398	3.2798133	Left	Insula	13
2	3,224	−10	−76	48	0.01004661	4.2205467	Left	Precuneus	7
2	3,224	−16	−76	42	0.00981178	4.1814437	Left	Precuneus	7
2	3,224	−6	−60	46	0.006709485	3.1047783	Left	Precuneus	7
aMCI>HC									
NA									
aMCI < HC									
1	6,928	−56	8	0	0.009969907	4.322146	Left	Superior temporal gyrus	22
1	6,928	−54	6	8	0.009410367	4.1647773	Left	Precentral gyrus	44
1	6,928	−52	18	2	0.008123473	3.4509804	Left	Precentral gyrus	44
1	6,928	−46	6	−6	0.00798626	3.422147	Left	Insula	13
1	6,928	−46	−2	−2	0.007434398	3.3515155	Left	Insula	13
2	4,128	−10	−76	48	0.01004661	4.335871	Left	Precuneus	7
2	4,128	−16	−76	42	0.00981178	4.297305	Left	Precuneus	7
2	4,128	−6	−60	46	0.006709485	3.1681263	Left	Precuneus	7
ReHo									
MCI>HC									
NA									
MCI < HC									
NA									
aMCI>HC									
NA									
aMCI < HC									
1	8,672	−8	44	−2	0.009139125	4.1076794	Left	Anterior cingulate	32
1	8,672	8	34	8	0.00801415	3.7818716	Right	Anterior cingulate	24
FC									
MCI>HC									
1	8,808	0	−12	7.5	0	2.2455866	Left	Thalamus	–
1	8,808	0	−7.5	16.5	0	2.3138068	Left	Thalamus	–
2	3,024	−10.5	12	7.5	0.002202077	2.4327567	Left	Caudate	–
3	2,936	−39	−33	18	0.007414908	3.4869988	Left	Superior temporal gyrus	41
3	2,936	−45	−27	15	0.007187633	3.3858383	Left	Insula	41
4	2,848	29	12	44	0.008558434	3.769974	Right	Middle frontal gyrus	6
4	2,848	24	14	45	0.009539967	4.119189	Right	Cingulate gyrus	32
MCI < HC									
1	2,480	−45	43.5	16.5	0.002824822	2.3455477	Left	Middle frontal gyrus	46
1	2,480	−45	42	27	0.007414908	3.4772985	Left	Superior frontal gyrus	9
aMCI < HC									
1	3,848	−34	24	−8	0.011685779	4.9532757	Left	Inferior frontal gyrus	47
aMCI>HC									
NA									

*BA, Brodmann area; ALE, anatomical/activation likelihood estimation; MCI, mild cognitive impairment; HC, healthy control; ALFF/fALFF, the amplitude of low-frequency fluctuation/fractional amplitude of low-frequency fluctuation; ReHo, regional homogeneity; FC, functional connectivity*.

#### Jackknife Sensitivity Analysis

A jackknife sensitivity analysis revealed that the decreased ALFF/fALFF in the left PreCG was the most robust data replicable in all 12 datasets. The decreased ALFF/fALFF in the left STG and insula remained highly replicable in at least 11 combinations of the datasets. The decreased ALFF/fALFF in the left precuneus remained significant in at least nine combinations of the datasets. The results are presented in the Supplementary Material.

## Discussion

To the best of our knowledge, this meta-analysis was the first to assess the functional integrity of the SN in MCI and aMCI. Compared with the healthy group, the specific abnormal brain regions in MCI were mainly located in the frontal lobe, including MFG, SFG, and PreCG; the limbic lobe, including cingulate gyrus; and the occipital lobe, including precuneus. Besides, there were also differences in the temporal lobe, including STG, and sub-lobar, including the thalamus and the caudate. Patients with aMCI demonstrated specific changes in the bilateral ACG and the left IFG.

### Specific Imaging Abnormal Changes in SN

Both patients with MCI and patients with aMCI showed decreased ALFF/fALFF in the left STG, left insula, left PreCG, and the precuneus. This indicated that the intrinsic neural activity of these regions had significant dysfunction in MCI and aMCI when compared with HCs (Zhao et al., 2014; Zhuang et al., 2019).

The STG is related to the cognition process and social interaction and plays an important role in the function of language (Li et al., 2017b). Dysfunction of the STG may result in the impairment of language and social skills in MCI. Localized changes of the STG were present after mindfulness practice for three months in subjects with MCI (Fam et al., 2020). The insula, especially the anterior insula, is a critical node of the SN which connects many areas including the prefrontal cortex and the parietal lobe and engages in the transformation between cognitive domains (Cerliani et al., 2012). A study by Na et al. reported that the MCI subgroup revealed focal activation in the left anterior insula and ACC after 24 sessions of computerized cognitive training (Na et al., 2018). The PreCG, which overlaps with the SMA, is involved in producing and controlling the movement (Cai et al., 2017). Abnormalities in the PreCG may affect learning and memorizing and show sluggish behavior; a positive correlation between the damage of this region and dysfunction of verbal short-term memory was reported in MCI (Bi et al., 2018; Sakurai et al., 2018). Increased blood oxygen level-dependent signal activation in the PreCG in MCI after daily supplementation for 16 weeks was reported by a clinical study that investigated the curative effect of blueberry supplementation (Boespflug et al., 2018). The precuneus is associated with mental imagery, source memory, and working memory (Utevsky et al., 2014). Decreased activity of neurons in the precuneus was shown to be connected with a steeper decline of global cognition function (Verfaillie et al., 2018). Patients in the early stages of AD have been shown to present hypoperfusion or metabolic reduction of the precuneus, and a positive correlation between the weaker function of the precuneus and lower MoCA score or lower working memory performance has been reported (Miners et al., 2016; Yokosawa et al., 2020). Therefore, when converting from health to MCI, the dysfunction of these brain regions would occur, which could be considered as the neuroimaging marker of MCI. Moreover, these brain regions can be regarded as the target for intervention treatment and follow-up care.

Our meta-analysis also observed increased FC in the thalamus, the caudate, and the left STG/insula in MCI when compared with HCs. The thalamus contributes to attentional control by high-fidelity information relay to cortical regions and shifting and sustaining functional interactions across cortical areas and is conventionally recognized to be affected by AD pathology (Halassa and Kastner, 2017). Pathological studies of thalamic involvement in AD demonstrated that nearly all thalamic nuclei got extracellular amyloid deposits, as well as the presence of thalamic volume loss (Ryan et al., 2013). The caudate contributes to behavior via activating correct action schemas and selecting opportune sub-goals and is associated with various higher neurological functions. A recent study that analyzed resting-state networks in prodromal AD also detected increased FC in the caudate in patients with positive biomarkers suggestive of incipient AD (Conwell et al., 2018). Previous studies had confirmed that the reduced volume of the caudate was significant with MCI-to-AD conversion (Madsen et al., 2010). Thus, we demonstrated that increased FC of the thalamus and the caudate in the SN, which may be considered as a compensated mechanism within the SN in MCI, can also be a specific marker of converting from health to MCI. The absence of this mechanism predicts the progress from MCI to dementia.

Besides, significantly increased FC and decreased ALFF/fALFF were simultaneously observed in the left STG/insula in MCI, compared with HCs. Studies have demonstrated that the ALFF/fALFF focuses on the intrinsic neural oscillation alterations, while the FC focuses on the connective relationship of the brain (Chen et al., 2016; Zhou et al., 2020). Therefore, the increased connectivity of the SN in these two areas may make up for deficits in their neuronal activity. Moreover, we hypothesized that further dysfunction of partial neurons of some brain regions would occur in subjects when converting to MCI. However, the other neurons of these brain regions could make some compensation by increasing their own activity or by connecting with others to maintain their cognitive state. Increased alteration in the STG/insula, as well as the increased FC in the left thalamus and the caudate, observed in this study could make up for the dysfunction to some extent, and the STG/insula might be the most susceptible one. As the disease progresses, the normal functional patterns are affected and partial compensatory is necessary.

With regard to ReHo, few studies have reported significant changes in the SN in MCI. An rs-fMRI study composing 30 MCI and 32 HCs revealed an increased alteration in the left precuneus in MCI (Wang et al., 2015), and other studies reported an increase in the left lingual gyrus or the right MFG, compared with HCs (Yuan X. et al., 2016; Luo et al., 2018). Some studies have demonstrated a reduction in changes in the right SG, the left MFG, or the left ACC (Zhang et al., 2012; Yuan B. et al., 2016). However, in this study, we did not observe consistent results from the ALE analysis. This could be explained by the fact that limited rs-fMRI studies focusing on the SN to investigate ReHo changes in MCI were available. Moreover, different stages of MCI may present different changes in the SN.

In terms of patients with aMCI, compared with HCs, besides consistent results of alterations in MCI, diminished ReHo was explicitly observed in the bilateral ACG, which indicated that local synchronization of the bilateral ACG was significantly declined in aMCI (Bai et al., 2008; Zhang et al., 2010). The ACG, in healthy and clinical individuals, plays a vital role in the conflict-monitoring process in attentional tasks (Borsa et al., 2018). Luks et al. reported that in the clinical populations, the atrophy of ACC and DLPFC in the left hemisphere was directly associated with poorer attentional control accuracy in patients with neurodegenerative diseases, compared with HCs (Luks et al., 2010). It is also imperative to note that studies have confirmed the impairment of endogenously orienting attention in patients with aMCI and AD. Furthermore, several studies combining MRI and SPECT have reported that aMCI converters can augment hypometabolism in ACG, when compared with subjects who did not convert to AD (Van Dam et al., 2013; McLaughlin et al., 2014). Therefore, the specific alteration of the ACG may help to distinguish aMCI from healthy elderly and even predict whether patients with aMCI will convert to AD.

### Salience Network Interaction With Other Networks

Reviewing the results of ALE analysis of the FC of the SN, interactions of the SN with DMN and ECN had been observed in MCI or aMCI. The key areas exhibiting interactions with DMN included the SFC and the ACC (Liao et al., 2010; Cunningham et al., 2017), and the ones showing interactions with ECN were mainly the SFG and the IFG (Xu et al., 2020). Recently, studies based on three large-scale functional brain networks or a triple-network model, the DMN, SN, and ECN, have been carried out (Joo et al., 2016; Li et al., 2019; Yu et al., 2019). The main functions of the three networks are not similar but they have anatomical coincident areas and co-activation. Regulating other networks might be a possible function of the SN, and dysregulated interactions among the three networks closely correlate with the cognitive dysfunction (Jilka et al., 2014; Liao et al., 2018).

The DMN, which is the main focus of neural-related rs-fMRI, is a task-negative network associated with information processing related to memory, emotion, and learning and contributes to support mind wandering during self-referential mental processing. The network is active when the individual is in the resting state (Esposito et al., 2018). The SN is coupling with the DMN, and the increased coupling within the two networks can be observed across cognitive tasks (Elton and Gao, 2015). Converging evidence indicates a linear relationship between the amyloid deposition and the dysfunction of DMN connectivity (Schultz et al., 2020). Previous studies concerning a deficit in DMN revealed a much more significant alteration in the AD group, thereby suggesting that the DMN had a progression of the connectivity deficit (Zhu et al., 2016). Consistent with previous reports, our results demonstrated that the SFG, which is one of the typical regions of the DMN, showed lower FC with the SN in MCI groups when compared with HCs (Liang et al., 2012). The ACC is the anterior part of the cingulate cortex (Torta et al., 2013). This brain region is the core one of the SN and is involved in integrating and coordinating information to guide behavior (Colangeli et al., 2016; Zeuner et al., 2016). However, the posterior part of the cingulate cortex (posterior cingulate cortex, PCC) is the core node of the DMN (Vogt, 2005; Cera et al., 2019). Studies have shown disrupted FC in the PCC in MCI when compared with HCs (Dillen et al., 2017). According to the posterior-to-anterior shift in aging theory, the cognitive performance is maintained by the recruitment of anterior regions when the function of the posterior region is impaired (Grady, 2012; Skouras et al., 2019). Compared with HCs, the increased FC of the ACC in the SN in MCI might be interpreted as a compensation mechanism to counter decay affected by impairments of posterior regions, thereby suggesting that the interaction among networks may be associated with a structural location.

The ECN governs executive functions, including problem-solving and working memory, and plays a key role in the regulation of cognition and the integration of memory and sensory information (Xu et al., 2020). Patients with MCI showed a significantly decreased FC, as well as increased FC, of the SN in the left MFG compared with HCs (He et al., 2014). The MFG is important for the execution of emotion regulation strategies (Japee et al., 2015). The executive functional impairment is considered to aggravate memory decline (Yuan B. et al., 2016). When the executive function of individuals was mildly affected, some compensatory changes were particularly necessary. This implied that impairment mechanism and compensation mechanism coexist among the SN-ECN connectivity in patients with MCI. Additionally, the IFG exhibited an aMCI-specific change for the SN-ECN connectivity in the FC in this study. The IFG, along with the hippocampus, drives encoding and retrieval of memory (Hampstead et al., 2016). This area also contributes to response inhibition, which is an important element in executive control (Jahanshahi et al., 2015). A study by He et al. reported significant differences in the internetwork FCs of the SN with the IFG in aMCI (He et al., 2014). Besides the SN-ECN connectivity, analyzing the FC of the DMN in aMCI revealed decreased FC between the DMN and the left IFG (Li et al., 2017c). Thus, the abnormality of the IFG could be considered as one of the major factors contributing to the pathological mechanism of aMCI. Studies have also reported the gradient change in the FC of the ECN from aMCI to AD (Zhu et al., 2016). This further supports the abnormality of FC as a biomarker for monitoring disease progression.

The FC-altered interactions between SN and other networks, such as DMN and ECN, were mainly present in the prefrontal lobe (SFG, MFG, and IFG) (Petrelli et al., 2020). Studies have shown that when compared with HCs, the perfusion in the frontal lobe was significantly lower in MCI (Zhang et al., 2019). A recent study reported that following 12 weeks of exercise training, there was an increase in the FC in the prefrontal lobe and insular lobe in MCI (Chirles et al., 2017). A meta-analysis for target identification in the non-invasive brain stimulation of AD and behavioral variant frontotemporal dementia demonstrated the core targets, including the MFG and SFG (Pievani et al., 2017). A systematic review of repetitive transcranial magnetic stimulation (rTMS) effects revealed that high-frequency rTMS, which targeted the left DLPFC, significantly improved the memory power (Chou et al., 2020). Thus, the prefrontal lobe, particularly the IFG in aMCI, can be a key target for intervention.

## Limitations

This meta-analysis had some limitations. First, the heterogeneity is the main limitation of this study and is due to the following four aspects: (1) data sources with different demographics of the subjects and acquisition parameters; (2) data quality, such as differences in motion correction and noise, affected the results; (3) data processing schemes including divergent preprocessing protocols, seed selection methods, and threshold settings; and (4) aberrant structural abnormalities, which were related to functional connectivity, were ignored in some studies. Second, besides the MCI group, we analyzed only the aMCI group. A meta-analysis based on naMCI was not conducted due to the inadequacy of the number of relative papers. Analysis of the subtype of MCI such as early MCI or later MCI is also neglected. Finally, we excluded the literature that was not published in English; hence, the data, which we have collected, might be incomplete.

## Clinical Implications

Although amounts of single studies have been done to determine the characteristics of the SN of patients with MCI and its subtype, making quantitative neuroimaging analysis is needed to summarize the neurobiology of the SN in MCI and aMCI. Uniform and precious results determine more exact targets for moderate interventions such as drug, transcranial direct current stimulation, rTMS, deep brain stimulation, and proper functional training. Core brain regions, such as STG/insula, the left MFG, and IFG, can be considered as potential targets in patients with MCI or aMCI. At the same time, these brain areas are also regarded as focuses for follow-up to understand the effect of interventions and the pathogenesis of diseases. To sum up, our results strongly demonstrated the specific characteristics of affected brain areas and provided new insights for targeted treatment and follow-up care.

## Conclusions

By conducting the ALE meta-analysis in patients with MCI and aMCI to identify the functional alterations about the SN, we have demonstrated the coexistence of impairment and compensation in the SN of patients with MCI, which is observed mainly in the SFG and the insula. Moreover, interactions of the SN with other networks, including changed the SN-DME connectivity and changed the SN-ECN connectivity, are also observed in MCI or aMCI. These findings can be a potential imaging biomarker for MCI or aMCI. Meanwhile, it has also provided a new insight in predicting the progression from a healthy state to MCI and novel targets for proper intervention to delay the further decline of cognitive function. Further research about MCI with comparisons between its subtypes needs to be carried out to explore more specific biomarkers for the predementia phase of AD.

## Data Availability Statement

The original contributions presented in the study are included in the article/Supplementary Material, further inquiries can be directed to the corresponding author/s.

## Author Contributions

YS designed the study under the guidance of XL and JC. YS and WX performed the study extraction, meta-analysis, and drafting of the manuscript. SC, GH, HG, CX, and WQ helped in literature search and data analyses. All authors contributed toward revising the manuscript and gave the final approval of the version to be published.

## Conflict of Interest

The authors declare that the research was conducted in the absence of any commercial or financial relationships that could be construed as a potential conflict of interest.

## Publisher's Note

All claims expressed in this article are solely those of the authors and do not necessarily represent those of their affiliated organizations, or those of the publisher, the editors and the reviewers. Any product that may be evaluated in this article, or claim that may be made by its manufacturer, is not guaranteed or endorsed by the publisher.
