# Supplementary material for: Functional MRI-Specific Alterations in Salience Network in Mild Cognitive Impairment: An ALE Meta-Analysis
Source: Front Aging Neurosci. 2021 Jul 26;13:695210. doi: 10.3389/fnagi.2021.695210 (PMC8350339; doi:10.3389/fnagi.2021.695210)
Supplement: Supplementary file 1 [file Data_Sheet_1.zip › Jackknife sensitivity analysis 695210.pdf]

Jackknife sensitivity analysis of the group of decreased ALFF/fALFF in MCI

All studies but…	the Left Superior Temporal Gyrus	the Left Precentral Gyrus	the Left Insula	the Left Precuneus
Cai, S.,2017[1]	√	√	√	√
Cha, J.,2015[2]	×	√	√	×
Jia, B.,2015[3]	√	√	√	√
Liang, P.,2014[4]	√	√	√	√
Liu, X.,2014[5]	√	√	√	√
Yang, L.,2018[6]	√	√	√	×
Zhao, Z.,2014[7]	√	√	×	√
Zhuang, L.,2019[8]	√	√	√	√
Zhuang, L.,2012[9]	√	√	√	√
Wang, Z.,2011[10]	√	√	√	√
Yin, C.,2014[11]	√	√	√	×
Li, Y.,2017[12]	√	√	√	√
Total	11 out of 12	12 out of 12	11 out of 12	9 out of 12

1 Cai, S. *et al.* Altered functional brain networks in amnestic mild cognitive impairment: a resting-state fMRI study. *Brain imaging and behavior* **11**, 619-631, doi:10.1007/s11682-016-9539-0 (2017).

2 Cha, J. *et al.* Assessment of Functional Characteristics of Amnestic Mild Cognitive Impairment and Alzheimer's Disease Using Various Methods of Resting-State FMRI Analysis. *BioMed research international* **2015**, 907464, doi:10.1155/2015/907464 (2015).

3 Jia, B. *et al.* The Effects of Acupuncture at Real or Sham Acupoints on the Intrinsic Brain Activity in Mild Cognitive Impairment Patients. *Evidence-based complementary and alternative medicine : eCAM* **2015**, 529675, doi:10.1155/2015/529675 (2015).

4 Liang, P. *et al.* Altered Amplitude of Low-frequency Fluctuations in Early and Late Mild Cognitive Impairment and Alzheimer's Disease. *Current Alzheimer research* **11**, 389-398, doi:10.2174/1567205011666140331225335 (2014).

5 Liu, X. *et al.* The association between TOMM40 gene polymorphism and spontaneous brain activity in amnestic mild cognitive impairment. *Journal of neurology* **261**, 1499-1507, doi:10.1007/s00415-014-7368-x (2014).

6 Yang, L. *et al.* Gradual Disturbances of the Amplitude of Low-Frequency Fluctuations (ALFF) and Fractional ALFF in Alzheimer Spectrum. *Frontiers in neuroscience* **12**, 975, doi:10.3389/fnins.2018.00975 (2018).

7 Zhao, Z. *et al.* Selective changes of resting-state brain oscillations in aMCI: an fMRI study using ALFF. *BioMed research international* **2014**, 920902, doi:10.1155/2014/920902 (2014).

8 Zhuang, L., Liu, X., Shi, Y., Liu, X. & Luo, B. Genetic Variants of PICALM rs541458 Modulate Brain Spontaneous Activity in Older Adults With Amnestic Mild Cognitive Impairment. *Frontiers in neurology* **10**, 494, doi:10.3389/fneur.2019.00494 (2019).

9 Zhuang, L. *et al.* Association of the interleukin 1 beta gene and brain spontaneous activity in amnestic mild cognitive impairment. *Journal of neuroinflammation* **9**, 263,

doi:10.1186/1742-2094-9-263 (2012).

10 Wang, Z. *et al.* Spatial patterns of intrinsic brain activity in mild cognitive impairment and alzheimer's disease: A resting-state functional MRI study. *Human brain mapping* **32**, 1720-1740, doi:10.1002/hbm.21140 (2011).

11 Yin, C. *et al.* Early morphological brain abnormalities in patients with amnestic mild cognitive impairment. *Translational Neuroscience* **5**, 253-259, doi:10.2478/s13380-014-0234-6 (2014).

12 Li, Y. *et al.* Frequency-Dependent Changes in the Amplitude of Low-Frequency Fluctuations in Mild Cognitive Impairment with Mild Depression. *Journal of Alzheimer's disease : JAD* **58**, 1175-1187, doi:10.3233/jad-161282 (2017).
