# Supplementary material for: Functional MRI-Specific Alterations in Salience Network in Mild Cognitive Impairment: An ALE Meta-Analysis
Source: Front Aging Neurosci. 2021 Jul 26;13:695210. doi: 10.3389/fnagi.2021.695210 (PMC8350339; doi:10.3389/fnagi.2021.695210)
Supplement: Supplementary file 1 [file Data_Sheet_1.zip › Quality assessment of included studies 695210.pdf]

Methodological quality assessment of the included case-control studies with the Newcastle Ottawa Scale											
		Selection				Comparability		Outcome			Score
**Number**	**Author, Year**	**Adequate Case Definition**	**Representative of Cases**	**Selection of Controls**	**Definition of Controls**	**Control For Important Factors**	**Additional Factors**	**Ascertainment of Exposure**	**Same Method of Ascertainment for Cases and Controls**	**Non-Response Rate**	**Total**
1	Cai, S.2017[1]	+	+	+	+	+	+	-	+	-	7
2	Cha, J.2015[2]	+	+	-	+	+	+	+	+	+	8
3	Jia, B.2015[3]	+	+	-	+	+	+	+	+	-	7
4	Liang, P.2014[4]	+	+	+	+	+	+	-	+	-	7
5	Liu, X.2014[5]	+	+	-	+	+	+	+	+	-	7
6	Li, Y.2017[6]	+	+	+	+	+	+	+	+	-	8
7	Ni, L.2016[7]	+	+	-	+	+	+	+	+	+	8
8	Wang, Z.2011[8]	+	-	+	+	+	+	+	+	-	7
9	Yang, L.2018[9]	+	+	+	+	+	+	+	+	-	8
10	Yin, C.2014[10]	-	+	+	-	+	+	-	+	-	5
11	Zhao, Z.2014[11]	+	+	+	+	+	+	-	+	-	7
12	Zhuang, L.2019[12]	+	+	+	+	+	+	+	+	-	8
13	Zhuang, L.2012[13]	+	-	+	+	+	+	+	+	-	7
14	Zhou, Q. H.2020[14]	+	+	+	+	+	+	+	+	-	8
15	Zhuang, L.2020[15]	+	+	-	+	-	+	+	+	-	6
16	Bai, F.2008[16]	+	-	-	+	+	+	+	+	-	6
17	Cai, S.2018[17]	+	+	+	+	+	+	-	+	-	7
18	Liu, Z.2014[18]	+	+	+	-	+	+	+	+	-	7
19	Luo, X.2018[19]	+	+	+	+	+	+	+	+	-	8

20	Min, J.2019[20]	+	-	+	+	+	+	-	+	-	6
21	Wang, Y.2015[21]	+	+	+	+	+	+	+	+	-	8
22	Yuan, X.2016[22]	+	+	-	-	+	+	-	+	-	5
23	Zhang, Z.2010[23]	+	-	-	+	+	+	-	+	-	5
24	Conwell, K.2018[24]	+	+	+	-	+	+	-	+	-	6
25	He, X.2014[25]	+	+	+	-	+	+	+	+	-	7
26	Liang, P.2012[26]	+	-	+	-	+	+	-	+	-	6
27	Li, X.2020[27]	+	+	-	+	+	+	+	+	-	7
28	Sarli, G.2021[28]	+	+	+	-	+	+	+	+	-	7
29	Yi, D.2015[29]	+	+	-	+	+	+	+	+	-	7
30	Zhu, H.2016[30]	+	+	-	+	+	+	-	+	-	6

NB: + met, - unmet or not applicable

1 Cai, S. *et al.* Altered functional brain networks in amnestic mild cognitive impairment: a resting-state fMRI study. *Brain imaging and behavior* **11**, 619-631, doi:10.1007/s11682-016-9539-0 (2017).

2 Cha, J. *et al.* Assessment of Functional Characteristics of Amnestic Mild Cognitive Impairment and Alzheimer's Disease Using Various Methods of Resting-State FMRI Analysis. *BioMed research international* **2015**, 907464, doi:10.1155/2015/907464 (2015).

3 Jia, B. *et al.* The Effects of Acupuncture at Real or Sham Acupoints on the Intrinsic Brain Activity in Mild Cognitive Impairment Patients. *Evidence-based complementary and alternative medicine : eCAM* **2015**, 529675, doi:10.1155/2015/529675 (2015).

4 Liang, P. *et al.* Altered amplitude of low-frequency fluctuations in early and late mild cognitive impairment and Alzheimer's disease. *Current Alzheimer research* **11**, 389-398, doi:10.2174/1567205011666140331225335 (2014).

5 Liu, X. *et al.* The association between TOMM40 gene polymorphism and spontaneous brain activity in amnestic mild cognitive impairment. *Journal of neurology* **261**, 1499-1507, doi:10.1007/s00415-014-7368-x (2014).

6 Li, Y. *et al.* Frequency-Dependent Changes in the Amplitude of Low-Frequency Fluctuations in Mild Cognitive Impairment with Mild Depression. *Journal of Alzheimer's disease : JAD* **58**, 1175-1187, doi:10.3233/jad-161282 (2017).

7 Ni, L. *et al.* Aberrant Spontaneous Brain Activity in Patients with Mild Cognitive Impairment and concomitant Lacunar Infarction: A Resting-State Functional

MRI Study. *Journal of Alzheimer's disease : JAD* **50**, 1243-1254, doi:10.3233/jad-150622 (2016).
8 Wang, Z. *et al.* Spatial patterns of intrinsic brain activity in mild cognitive impairment and alzheimer's disease: A resting-state functional MRI study. *Human brain mapping* **32**, 1720-1740, doi:10.1002/hbm.21140 (2011).
9 Yang, L. *et al.* Gradual Disturbances of the Amplitude of Low-Frequency Fluctuations (ALFF) and Fractional ALFF in Alzheimer Spectrum. *Frontiers in neuroscience* **12**, 975, doi:10.3389/fnins.2018.00975 (2018).
10 Yin, C. *et al.* Early morphological brain abnormalities in patients with amnestic mild cognitive impairment. *Translational Neuroscience* **5**, 253-259, doi:10.2478/s13380-014-0234-6 (2014).
11 Zhao, Z. *et al.* Selective changes of resting-state brain oscillations in aMCI: an fMRI study using ALFF. *BioMed research international* **2014**, 920902, doi:10.1155/2014/920902 (2014).
12 Zhuang, L., Liu, X., Shi, Y., Liu, X. & Luo, B. Genetic Variants of PICALM rs541458 Modulate Brain Spontaneous Activity in Older Adults With Amnestic Mild Cognitive Impairment. *Frontiers in neurology* **10**, 494, doi:10.3389/fneur.2019.00494 (2019).
13 Zhuang, L. *et al.* Association of the interleukin 1 beta gene and brain spontaneous activity in amnestic mild cognitive impairment. *Journal of neuroinflammation* **9**, 263, doi:10.1186/1742-2094-9-263 (2012).
14 Zhou, Q. H., Wang, K., Zhang, X. M., Wang, L. & Liu, J. H. Differential Regional Brain Spontaneous Activity in Subgroups of Mild Cognitive Impairment. *Frontiers in human neuroscience* **14**, 2, doi:10.3389/fnhum.2020.00002 (2020).
15 Zhuang, L. *et al.* Aggregation of Vascular Risk Factors Modulates the Amplitude of Low-Frequency Fluctuation in Mild Cognitive Impairment Patients. *Frontiers in aging neuroscience* **12**, 604246, doi:10.3389/fnagi.2020.604246 (2020).
16 Bai, F. *et al.* Default-mode network activity distinguishes amnestic type mild cognitive impairment from healthy aging: A combined structural and resting-state functional MRI study. *Neuroscience letters* **438**, 111-115, doi:10.1016/j.neulet.2008.04.021 (2008).
17 Cai, S. *et al.* Differentiated Regional Homogeneity in Progressive Mild Cognitive Impairment: A Study With Post Hoc Label. *American journal of Alzheimer's disease and other dementias* **33**, 373-384, doi:10.1177/1533317518778513 (2018).
18 Liu, Z. *et al.* Exploring the patterns of acupuncture on mild cognitive impairment patients using regional homogeneity. *PloS one* **9**, doi:10.1371/journal.pone.0099335 (2014).
19 Luo, X. *et al.* Alteration of regional homogeneity and white matter hyperintensities in amnestic mild cognitive impairment subtypes are related to cognition and CSF biomarkers. *Brain imaging and behavior* **12**, 188-200, doi:10.1007/s11682-017-9680-4 (2018).
20 Min, J., Zhou, X. X., Zhou, F., Tan, Y. & Wang, W. D. A study on changes of the resting-state brain function network in patients with amnestic mild cognitive

impairment. *Brazilian journal of medical and biological research = Revista brasileira de pesquisas medicas e biologicas* **52**, e8244, doi:10.1590/1414-431x20198244 (2019).

21 Wang, Y. *et al.* Using regional homogeneity to reveal altered spontaneous activity in patients with mild cognitive impairment. *BioMed research international* **2015**, 807093, doi:10.1155/2015/807093 (2015).

22 Yuan, X. *et al.* Regional homogeneity changes in amnestic mild cognitive impairment patients. *Neuroscience letters* **629**, 1-8, doi:10.1016/j.neulet.2016.06.047 (2016).

23 Zhang, Z. *et al.* Alteration of resting brain function by genetic variation in angiotensin converting enzyme in amnestic-type mild cognitive impairment of Chinese Han. *Behavioural brain research* **208**, 619-625, doi:10.1016/j.bbr.2010.01.008 (2010).

24 Conwell, K. *et al.* Test-retest variability of resting-state networks in healthy aging and prodromal Alzheimer's disease. *NeuroImage. Clinical* **19**, 948-962, doi:10.1016/j.nicl.2018.06.016 (2018).

25 He, X. *et al.* Abnormal salience network in normal aging and in amnestic mild cognitive impairment and Alzheimer's disease. *Human brain mapping* **35**, 3446-3464, doi:10.1002/hbm.22414 (2014).

26 Liang, P., Wang, Z., Yang, Y. & Li, K. Three subsystems of the inferior parietal cortex are differently affected in mild cognitive impairment. *Journal of Alzheimer's disease : JAD* **30**, 475-487, doi:10.3233/jad-2012-111721 (2012).

27 Li, X. *et al.* Changes in Brain Function Networks in Patients With Amnestic Mild Cognitive Impairment: A Resting-State fMRI Study. *Frontiers in neurology* **11**, 554032, doi:10.3389/fneur.2020.554032 (2020).

28 Sarli, G. *et al.* Regional Strength of Large-Scale Functional Brain Networks Is Associated with Regional Volumes in Older Adults and in Alzheimer's Disease. *Brain connectivity*, doi:10.1089/brain.2020.0899 (2021).

29 Yi, D. *et al.* Differences in functional brain connectivity alterations associated with cerebral amyloid deposition in amnestic mild cognitive impairment. *Frontiers in aging neuroscience* **7**, 15, doi:10.3389/fnagi.2015.00015 (2015).

30 Zhu, H. *et al.* Changes of intranetwork and internetwork functional connectivity in Alzheimer's disease and mild cognitive impairment. *Journal of neural engineering* **13**, 046008, doi:10.1088/1741-2560/13/4/046008 (2016).
