# Supplementary material for: Functional MRI-Specific Alterations in Salience Network in Mild Cognitive Impairment: An ALE Meta-Analysis
Source: Front Aging Neurosci. 2021 Jul 26;13:695210. doi: 10.3389/fnagi.2021.695210 (PMC8350339; doi:10.3389/fnagi.2021.695210)
Supplement: Supplementary file 1 [file Data_Sheet_1.zip › Seeds of included studies 695210.pdf]

1. He, X. *et al.* Abnormal salience network in normal aging and in amnestic mild cognitive impairment and Alzheimer's disease. *Human brain mapping* **35**, 3446-3464, doi:10.1002/hbm.22414 (2014).

The SN is identified using independent component analysis, whereas the CEN and DMN are identified using ROI-based functional connectivity analysis.

- Regions of the SN: bilateral dACC/MPFC; bilateral frontoinsular cortex; bilateral DLPFC
- ROIs of the CEN: the left DLPFC (MNI: 248, 34, 34), the right DLPFC (MNI coordinates: 48, 40, 30), the left PPC (MNI: 236, 244, 46), the right PPC (MNI: 42, 242, 48)
- ROIs of the DMN: the VMPFC (MNI: 22, 54, 26), the left PCC (MNI: 26, 248, 32), the right PCC (MNI: 10, 252, 28)

2. Liang, P., Wang, Z., Yang, Y. & Li, K. Three subsystems of the inferior parietal cortex are differently affected in mild cognitive impairment. *Journal of Alzheimer's disease : JAD* **30**, 475-487, doi:10.3233/jad-2012-111721 (2012).

- ROIs of the DMN: the angular gyrus
- ROIs of the CEN: the intraparietal sulcus
- ROIs of the SN: the supramarginal gyrus

3. Sarli, G. et al. Regional Strength of Large-Scale Functional Brain Networks Is Associated with Regional Volumes in Older Adults and in Alzheimer's Disease. Brain connectivity, doi:10.1089/brain.2020.0899 (2021).

Independent component analysis was conducted. The number of components to compute was set to twenty. The twenty resulting group-level maps were visually inspected by three independent raters to shortlist and detect those that represented large-scale functional brain networks of interest.

- ROIs of the DMN: the PCC, bilateral inferior parietal lobule
- ROIs of the SN: the ACC, bilateral insula
- ROIs of the SMN: bilateral precentral gyrus, bilateral postcentral gyrus

4. Zhu, H. *et al.* Changes of intranetwork and internetwork functional connectivity in Alzheimer's disease and mild cognitive impairment. *Journal of neural engineering* **13**, 046008, doi:10.1088/1741-2560/13/4/046008 (2016)

- ROIs of the DMN: the PCC (MNI:1, -55, 17)
- ROIs of the DAN: the intraparietal sulcus (MNI: -27, -51, 57; 24, -56, 55)
- ROIs of the ECN: bilateral DLPFC (MNI: 44, 36, 20; -32, 44, 16)
- ROIs of the SN: bilateral anterior insula (MNI: 38, 26, -10; -32, 24, -10)

Note: ACC: anterior cingulate cortex, dACC/MPFC: dorsal anterior cingulate cortex/medial prefrontal cortex, DLPFC: dorsolateral prefrontal cortex, PPC: posterior parietal cortex, PCC: posterior cingulate cortex, VMPFC: ventromedial prefrontal cortex; DMN: the default mode, DAN: the dorsal attention network, ECN: the executive control network, SN: the salience network; SMN: the sensorimotor network
